# Analytical framing of Amyloid beta monoclonal antibody trials in Alzheimer's disease can obscure clinical effects

**DOI:** 10.1002/alz.71646

**Published:** 2026-07-28

**Authors:** Betty M. Tijms, Argonde C. van Harten, Maria C. Carillo, Philip Scheltens, Everard G. B. Vijverberg

**Affiliations:** ^1^ Alzheimer Center Amsterdam, Neurology Vrije Universiteit Amsterdam, Amsterdam UMC location VUmc Amsterdam the Netherlands; ^2^ Neurodegeneration Amsterdam Neuroscience Amsterdam the Netherlands; ^3^ Alzheimer's Association Chicago Illinois USA; ^4^ EQT Life Sciences Amsterdam the Netherlands

**Keywords:** Alzheimer's disease, meta‐analysis, monoclonal anti‐amyloid antibodies, trials

## Abstract

Cochrane Reviews are widely regarded as the gold standard for evidence synthesis, yet analytical framing influences interpretation. A recent Cochrane review of amyloid beta–targeting monoclonal antibodies pools mechanistically and biologically heterogenous interventions. These antibodies differ in biological targets and clinical effects, making a class‐level estimate difficult to interpret. Pooling effects across these studies risks obscuring meaningful differences between interventions and may produce conclusions that do not reflect the underlying data, with important consequences for the evaluation of disease‐modifying therapies in Alzheimer's disease.

## Highlights

- Amyloid beta monoclonal antibodies differ in targets and clinical effects and are not interchangeable.
- High heterogeneity between studies limits interpretation of pooled treatment effects.
- Meta‐analyses are not immune to analytical framing, which can shape conclusions on disease‐modifying therapies.

1

Cochrane Reviews are widely regarded as the gold standard for synthesizing medical evidence, reflecting their rigorous methodological standards and emphasis on transparency. Consequently, their conclusions carry substantial weight for clinicians, regulators, patients, and the wider public.

Although Cochrane's author guidelines are intended to maximize methodological rigour and consistency, they are not immune to author bias or inappropriate analytical decisions (and therefore conclusions). The recent review by Nonino and colleagues on amyloid beta (Aβ) targeting monoclonal antibodies for mild cognitive impairment (MCI) and mild dementia due to Alzheimer's disease^1^ illustrates how framing can influence interpretation, with important implications for policy and clinical decisions surrounding the adoption of Alzheimer's disease‐modifying therapies.

In this review, clinically and mechanistically heterogeneous interventions are collapsed into a single analytical category. Yet, as acknowledged in the background section, these antibodies differ fundamentally in their targets, ranging from monomeric Aβ to distinct aggregated and plaque‐associated species associated with later stages of amyloid pathology. These differences translate into different pharmacodynamic profiles, including variation in plaque clearance, rates of clinical effects, and risk of amyloid‐related imaging abnormalities (ARIA). Therefore, regarding these antibodies as interchangeable interventions would be analogous to testing a vacuum, sponge, mop, and industrial degreaser on different types of dirt (dust, liquid spills, and hardened grease) and then concluding that cleaning is ineffective.

This conceptual issue indicates that the scope of the review question itself is overly broad. Chapter 2 of the Cochrane Handbook states that “the first and most important decision in preparing a systematic review is to determine its focus” and that “well‐formulated questions will guide many aspects of the review process, including determining eligibility criteria, searching for studies, collecting data from studies, structuring the syntheses and presenting the findings.”^2^ Thus, the Handbook explicitly recognizes that framing choices determine the interpretability of subsequent analyses, which ultimately may inform clinical and healthcare decision‐making. In the review by Nonino et al., interventions with different mechanisms of action, degrees of target engagement, and clinical effects are treated as a single therapeutic class, despite important biological and clinical differences. Although inclusion of negative trials is not inherently problematic, interventions should be stratified according to the mechanism of action and degree of target engagement. Such an approach would help clinicians and policymakers distinguish between mechanistically distinct therapeutic approaches and identify the most promising molecular targets. The pooled estimate provided in the review no longer addresses the clinically relevant question faced by clinicians and policymakers, namely whether interventions that effectively engage and clear pathological amyloid species have clinical effects. The PICO framework (which stand for and defines the P = Patient, Populations or Problem, I = Intervention, C = Comparison and O = Outcome) of this review should therefore be reconsidered by the authors.

The fundamental differences between these interventions are reflected in the results. The reported heterogeneity (indicated by the statistic I^2^, which quantifies the percentage of total variation between studies in a meta‐analysis that is due to true heterogeneity) for Clinical Dementia Rating–Sum of Boxes (CDR‐SB), often the primary outcome in Phase 3 trials, was substantial (I^2^ = 67% across effects and I^2^ = 76.7% for subgroup differences). These values fall within the range that the Cochrane Handbook characterizes as substantial (50%–90%) to considerable (75%–100%) statistical heterogeneity,^3^ indicating that much of the observed variation in effect sizes reflects *real* differences between interventions rather than random sampling error. Under such conditions, a pooled estimate is unlikely to represent any individual intervention. Chapter 10 of the Cochrane Handbook explicitly advises that under conditions of substantial heterogeneity, it may be misleading to quote an average intervention effect and that a systematic review does not need to contain a meta‐analysis.^3^ Nevertheless, the authors proceed to pool these interventions and subsequently conclude that the resulting effect is not clinically relevant.

In view of these conceptual and statistical issues, the certainty language overreaches. The abstract and plain‐language summary present a strong “no meaningful benefit” message that is likely to influence clinical and policy decisions. However, a pooled estimate derived from mechanistically incompatible trials does not provide a reliable basis for class‐level conclusions in either direction. The review further suggests that the field should move away from amyloid entirely, a statement that is not supported by the structure of the underlying data. However, the absence of effect for some agents does not logically imply failure of the underlying biological mechanism.

The review also mischaracterizes the populations enrolled in most trials. Recent trials (including those of aducanumab, lecanemab, and donanemab) enrolled only participants with biomarker‐confirmed amyloid pathology. However, subgroup analyses are framed as though amyloid‐negative participants may have been included in those studies. Similarly, cerebrovascular comorbidities were generally addressed through pre‐specified exclusion criteria in trials, contrary to the impression given by the review. Such inconsistencies increase the risk of misinterpretation.

Further issues include, among others, duplicate referencing of the same trials (e.g., references 97 and 111, 96 and 110), omission of results from relevant studies such as the GRADUATE trials for gantenerumab, misstating that solanezumab effectively cleared amyloid plaques from the brain, reporting different sample sizes than the intention to treat (ITT) population for the donanemab result, and presentation of progression risks in mild cognitive impairment (MCI) without stratification by amyloid, despite its strong influence on progression. For example, 3‐year progression to Alzheimer's dementia is about 5% in MCI with normal biomarkers, compared with 22% in amyloid‐positive MCI and 59% when both amyloid and tau are abnormal,^4^ an increased risk that is consistent across studies.^5^, ^6^

Taken together, the conclusions reflect the framing of the analysis rather than the structure of the underlying data. The result is an apparently robust but potentially misleading conclusion: Less a synthesis of evidence than an artifact of how that evidence is framed. In this respect the analysis recalls the myth of Procrustes: heterogenous evidence is forced into a single analytical frame, with differences removed to achieve uniformity. Cochrane's authority rests on methodological rigor. However, in this instance, the analysis appears to rely more on that authority than is justified by the underlying data.

## CONFLICT OF INTEREST STATEMENT

The authors declare no conflicts of interest. Author disclosures are available in the Supporting Information.

## Supporting information




**Supporting Information**: alz71646‐Sup‐0001‐ICMJE DISCLOSURE FORM_merged.pdf
