# Supplementary material for: Analytical framing of Amyloid beta monoclonal antibody trials in Alzheimer's disease can obscure clinical effects
Source: Alzheimers Dement. 2026 Jul 28;22(7):e71646. doi: 10.1002/alz.71646 (PMC13408426; doi:10.1002/alz.71646)
Supplement: Supplementary file 1 — Supporting Information: alz71646‐Sup‐0001‐ICMJE DISCLOSURE FORM_merged.pdf [file ALZ-22-e71646-s001.pdf]

ICMJE DISCLOSURE FORM

**Date:**	5/19/2026
**Your Name:**	Betty Tijms
**Manuscript Title:**	Analytical framing of amyloid-β monoclonal antibody trials in Alzheimer's disease can obscure clinical effects
**Manuscript Number (if known):**	ADJ-D-26-01453

In the interest of transparency, we ask you to disclose all relationships/activities/interests listed below that are related to the conte of your manuscript. "Related" means any relation with for-profit or not-for-profit third parties whose interests may be affected b the content of the manuscript. Disclosure represents a commitment to transparency and does not necessarily indicate a bias. If yo are in doubt about whether to list a relationship/activity/interest, it is preferable that you do so.

The author's relationships/activities/interests should be defined broadly. For example, if your manuscript pertains to the epidemiology of hypertension, you should declare all relationships with manufacturers of antihypertensive medication, even if tha medication is not mentioned in the manuscript.

In item #1 below, report all support for the work reported in this manuscript without time limit. For all other items, the time fram for disclosure is the past 36 months.

		**Name all entities with whom you have this relationship or indicate none (add rows as needed)**	**Specifications/Comments (e.g., if payments were made to you or to your institution)**
		**Time frame: Since the initial planning of the work**	
**1**	All support for the present manuscript (e.g., funding, provision of study materials, medical writing, article processing charges, etc.) **No time limit for this item.**	☐ **None**	
		ZonMW VIDI #09150171910068	
		ERC DecipherAD #101171721	
			Click the tab key to add additional rows.
		**Time frame: past 36 months**	
**2**	Grants or contracts from any entity (if not indicated in item #1 above).	☐ **None**	
		ZonMW TAP-dementia #10510032120003	
		Amsterdam Cohort Hub as part of the Sector Plan 'Accelerating Health' of the Dutch Ministry of Education, Culture and Science.	
		Merck research contract	Payments made to my institution
		Sanofi research contract	Payments made to my institution

		Name all entities with whom you have this relationship or indicate none (add rows as needed)	Specifications/Comments (e.g., if payments were made to you or to your institution)
		Eisai research contract	Payments made to my institution
		Roche research contract	Payments made to my institution
3	Royalties or licenses	☒ None	
4	Consulting fees	☒ None	
5	Payment or honoraria for lectures, presentations, speakers bureaus, manuscript writing or educational events	☐ None	
		Novo Nordisk	Payments made to my institution
		Sanofi	Payments made to my institution
6	Payment for expert testimony	☒ None	
7	Support for attending meetings and/or travel	☐ None	
		Novo Nordisk	Payments made to my institution
		Sanofi	Payments made to my institution
		ADDF	Payments made to my institution
8	Patents planned, issued or pending	☐ None	
		Co-inventor on patent of CSF proteomic subtypes published under number US2022196683A1	Owner Stichting VUmc

		Name all entities with whom you have this relationship or indicate none (add rows as needed)	Specifications/Comments (e.g., if payments were made to you or to your institution)
9	Participation on a Data Safety Monitoring Board or Advisory Board	☐ None	
		Roche	Payments made to my institution
10	Leadership or fiduciary role in other board, society, committee or advocacy group, paid or unpaid	☒ None	
11	Stock or stock options	☒ None	
12	Receipt of equipment, materials, drugs, medical writing, gifts or other services	☒ None	
13	Other financial or non-financial interests	☒ None	

Please place an "X" next to the following statement to indicate your agreement:

☒ I certify that I have answered every question and have not altered the wording of any of the questions on this form.

ICMJE DISCLOSURE FORM

**Date:**	5/20/2026
**Your Name:**	Argonde van Harten
**Manuscript Title:**	Analytical framing of amyloid-β monoclonal antibody trials in Alzheimer's disease can obscure clinical effects
**Manuscript Number (if known):**	ADJ-D-26-01453

In the interest of transparency, we ask you to disclose all relationships/activities/interests listed below that are related to the conte[illegible] of your manuscript. "Related" means any relation with for-profit or not-for-profit third parties whose interests may be affected b[illegible] the content of the manuscript. Disclosure represents a commitment to transparency and does not necessarily indicate a bias. If yo[illegible] are in doubt about whether to list a relationship/activity/interest, it is preferable that you do so.

The author's relationships/activities/interests should be defined broadly. For example, if your manuscript pertains to the epidemiology of hypertension, you should declare all relationships with manufacturers of antihypertensive medication, even if tha[illegible] medication is not mentioned in the manuscript.

In item #1 below, report all support for the work reported in this manuscript without time limit. For all other items, the time fram[illegible] for disclosure is the past 36 months.

		**Name all entities with whom you have this relationship or indicate none (add rows as needed)**	**Specifications/Comments (e.g., if payments were made to you or to your institution)**
**Time frame: Since the initial planning of the work**			
**1**	All support for the present manuscript (e.g., funding, provision of study materials, medical writing, article processing charges, etc.) **No time limit for this item.**	☒ **None**	
			Click the tab key to add additional rows.
**Time frame: past 36 months**			
**2**	Grants or contracts from any entity (if not indicated in	☐ **None**	
		Alzheimer Nederland	Payment made to institution
		ZonMW (TAP dementia project)	Payment made to institution
		Health Holland (Trinitas AlzTest)	Payment made to institution

		Name all entities with whom you have this relationship or indicate none (add rows as needed)	Specifications/Comments (e.g., if payments were made to you or to your institution)
	item #1 above).		
3	Royalties or licenses	☒ None	
4	Consulting fees	☐ None	
		Eli Lilly educational advisory board 2024	Payment made to insittution
5	Payment or honoraria for lectures, presentations, speakers bureaus, manuscript writing or educational events	☒ None	
6	Payment for expert testimony	☒ None	
7	Support for attending meetings and/or travel	☒ None	
8	Patents planned, issued or pending	☒ None	

		Name all entities with whom you have this relationship or indicate none (add rows as needed)	Specifications/Comments (e.g., if payments were made to you or to your institution)
9	Participation on a Data Safety Monitoring Board or Advisory Board	☒ None	
10	Leadership or fiduciary role in other board, society, committee or advocacy group, paid or unpaid	☒ None	
11	Stock or stock options	☒ None	
12	Receipt of equipment, materials, drugs, medical writing, gifts or other services	☒ None	
13	Other financial or non-financial interests	☒ None	

Please place an "X" next to the following statement to indicate your agreement:

☒ I certify that I have answered every question and have not altered the wording of any of the questions on this form.

ICMJE DISCLOSURE FORM

**Date:**	5/19/2026
**Your Name:**	Maria Carrillo
**Manuscript Title:**	Analytical framing of amyloid-β monoclonal antibody trials in Alzheimer's disease can obscure clinical effects
**Manuscript Number (if known):**	ADJ-D-26-01453

In the interest of transparency, we ask you to disclose all relationships/activities/interests listed below that are related to the conte of your manuscript. "Related" means any relation with for-profit or not-for-profit third parties whose interests may be affected by the content of the manuscript. Disclosure represents a commitment to transparency and does not necessarily indicate a bias. If yo are in doubt about whether to list a relationship/activity/interest, it is preferable that you do so.

The author's relationships/activities/interests should be defined broadly. For example, if your manuscript pertains to the epidemiology of hypertension, you should declare all relationships with manufacturers of antihypertensive medication, even if tha medication is not mentioned in the manuscript.

In item #1 below, report all support for the work reported in this manuscript without time limit. For all other items, the time fram for disclosure is the past 36 months.

		**Name all entities with whom you have this relationship or indicate none (add rows as needed)**	**Specifications/Comments (e.g., if payments were made to you or to your institution)**
		**Time frame: Since the initial planning of the work**	
**1**	All support for the present manuscript (e.g., funding, provision of study materials, medical writing, article processing charges, etc.) **No time limit for this item.**	☐ **None**	
		Alzheimer's Association	
		National Institutes of Health	
			Click the tab key to add additional rows.
		**Time frame: past 36 months**	
**2**	Grants or contracts from any entity (if not indicated in	☐ **None**	
		NIA and CDC	

		Name all entities with whom you have this relationship or indicate none (add rows as needed)	Specifications/Comments (e.g., if payments were made to you or to your institution)
	item #1 above).		
3	Royalties or licenses	☒ None	
4	Consulting fees	☒ None	
5	Payment or honoraria for lectures, presentations, speakers bureaus, manuscript writing or educational events	☒ None	
6	Payment for expert testimony	☒ None	
7	Support for attending meetings and/or travel	☐ None	
		Full time employee of the Alzheimer's Association; all travel covered by my employer	
8	Patents planned, issued or pending	☒ None	

		Name all entities with whom you have this relationship or indicate none (add rows as needed)	Specifications/Comments (e.g., if payments were made to you or to your institution)
9	Participation on a Data Safety Monitoring Board or Advisory Board	☐ None	
		NIA and NINDS funded initiatives including ADSP	
10	Leadership or fiduciary role in other board, society, committee or advocacy group, paid or unpaid	☐ None	
		GHR Foundation, board	
		American Heart Association, Research Committee (unpaid), no longer active	
11	Stock or stock options	☒ None	
12	Receipt of equipment, materials, drugs, medical writing, gifts or other services	☒ None	
13	Other financial or non-financial interests	☐ None	
		Full time employee of the Alzheimer's Association	
		Daughter is a neuroscience graduate student at USC	

Please place an "X" next to the following statement to indicate your agreement:

☒ I certify that I have answered every question and have not altered the wording of any of the questions on this form.

ICMJE DISCLOSURE FORM

**Date:**	5/19/2026
**Your Name:**	Philip Scheltens
**Manuscript Title:**	Analytical framing of amyloid-β monoclonal antibody trials in Alzheimer's disease can obscure clinical effects
**Manuscript Number (if known):**	ADJ-D-26-01453

In the interest of transparency, we ask you to disclose all relationships/activities/interests listed below that are related to the conte of your manuscript. "Related" means any relation with for-profit or not-for-profit third parties whose interests may be affected by the content of the manuscript. Disclosure represents a commitment to transparency and does not necessarily indicate a bias. If yo are in doubt about whether to list a relationship/activity/interest, it is preferable that you do so.

The author's relationships/activities/interests should be defined broadly. For example, if your manuscript pertains to the epidemiology of hypertension, you should declare all relationships with manufacturers of antihypertensive medication, even if tha medication is not mentioned in the manuscript.

In item #1 below, report all support for the work reported in this manuscript without time limit. For all other items, the time fram for disclosure is the past 36 months.

		Name all entities with whom you have this relationship or indicate none (add rows as needed)	Specifications/Comments (e.g., if payments were made to you or to your institution)
**Time frame: Since the initial planning of the work**			
**1**	All support for the present manuscript (e.g., funding, provision of study materials, medical writing, article processing charges, etc.) **No time limit for this item.**	☒ **None**	
			Click the tab key to add additional rows.
**Time frame: past 36 months**			
**2**	Grants or contracts from any entity (if not indicated in	☒ **None**	

		Name all entities with whom you have this relationship or indicate none (add rows as needed)	Specifications/Comments (e.g., if payments were made to you or to your institution)
	item #1 above).		
3	Royalties or licenses	☒ None	
4	Consulting fees	☐ None	
		NOVO Nordisk	Paid to university
5	Payment or honoraria for lectures, presentations, speakers bureaus, manuscript writing or educational events	☒ None	
6	Payment for expert testimony	☒ None	
7	Support for attending meetings and/or travel	☒ None	
8	Patents planned, issued or pending	☒ None	

		Name all entities with whom you have this relationship or indicate none (add rows as needed)	Specifications/Comments (e.g., if payments were made to you or to your institution)
9	Participation on a Data Safety Monitoring Board or Advisory Board	☐ None	
		Chair DSMB Retain Study (J&J)	
		Member DSMB ImmunoBrain	
10	Leadership or fiduciary role in other board, society, committee or advocacy group, paid or unpaid	☒ None	
11	Stock or stock options	☐ None	
		Stocks and stock options in EQT AB	
12	Receipt of equipment, materials, drugs, medical writing, gifts or other services	☒ None	
13	Other financial or non-financial interests	☐ None	
		Full time employee of EQT Life Sciences	

Please place an "X" next to the following statement to indicate your agreement:

☒ I certify that I have answered every question and have not altered the wording of any of the questions on this form.

ICMJE DISCLOSURE FORM

Date: 5/19/2026

Your Name: E.G.B. Vijverberg

Manuscript Title: Analytical framing of amyloid-β monoclonal antibody trials in Alzheimer's disease can obscure clinical effects

Manuscript Number (if known): ADJ-D-26-01453

In the interest of transparency, we ask you to disclose all relationships/activities/interests listed below that are related to the conte of your manuscript. "Related" means any relation with for-profit or not-for-profit third parties whose interests may be affected by the content of the manuscript. Disclosure represents a commitment to transparency and does not necessarily indicate a bias. If yo are in doubt about whether to list a relationship/activity/interest, it is preferable that you do so.

The author's relationships/activities/interests should be defined broadly. For example, if your manuscript pertains to the epidemiology of hypertension, you should declare all relationships with manufacturers of antihypertensive medication, even if tha medication is not mentioned in the manuscript.

In item #1 below, report all support for the work reported in this manuscript without time limit. For all other items, the time fram for disclosure is the past 36 months.

		Name all entities with whom you have this relationship or indicate none (add rows as needed)	Specifications/Comments (e.g., if payments were made to you or to your institution)
**Time frame: Since the initial planning of the work**			
1	All support for the present manuscript (e.g., funding, provision of study materials, medical writing, article processing charges, etc.) **No time limit for this item.**	☒ **None**	
			Click the tab key to add additional rows.
**Time frame: past 36 months**			
2	Grants or contracts from any entity (if not indicated in	☐ **None**	
		Within his university affiliation he is PI of studies of DIAN, AC immune, Alnylam, CogRX therapeutics, New Amsterdam Pharma, Janssen, UCB, Roche, Vivoryon, ImmunoBrain, GSK, MSD, Biogen, Alector, Eli Lilly, AriBio Fuij Film Toyama, GemVax. Co-founder van het CANDIDATE Center Amsterdam UMC and managing director of ADORE.	

		Name all entities with whom you have this relationship or indicate none (add rows as needed)	Specifications/Comments (e.g., if payments were made to you or to your institution)
	item #1 above).	Scientific projects with the Dutch Soccer Association (KNVB	
3	Royalties or licenses	☒ None	
4	Consulting fees	☐ None	
		Dr Vijverberg has received consultancy fees (paid to the university) for New Amsterdam Pharma, Treeway, ReMynd, Vivoryon, Biogen, Vigil Neuroscience, ImmunoBrain Checkpoint, Muna Therapeutics, Esai, Eli Lilly, CogRX, Therini, UCB and Roche.	
5	Payment or honoraria for lectures, presentations, speakers bureaus, manuscript writing or educational events	☐ None	
		From Lilly and Biogen	
6	Payment for expert testimony	☒ None	
7	Support for attending meetings and/or travel	☐ None	
		Lilly and Biogen	

		Name all entities with whom you have this relationship or indicate none (add rows as needed)	Specifications/Comments (e.g., if payments were made to you or to your institution)
8	Patents planned, issued or pending	☒ **None**	
9	Participation on a Data Safety Monitoring Board or Advisory Board	☒ **None**	
10	Leadership or fiduciary role in other board, society, committee or advocacy group, paid or unpaid	☒ **None**	
11	Stock or stock options	☒ **None**	
12	Receipt of equipment, materials, drugs, medical writing, gifts or other services	☒ **None**	
13	Other financial or non-financial interests	☒ **None**	

Please place an "X" next to the following statement to indicate your agreement:

☒ I certify that I have answered every question and have not altered the wording of any of the questions on this form.
